# Bridging radiotherapy before anti-CD19 CAR T-cell therapy for Large B-cell lymphoma – results from a single-center study

**DOI:** 10.1186/s13014-026-02822-z

**Published:** 2026-03-31

**Authors:** Sebastian M. Stolz, Camilla von Wachter, Jonas Willmann, Max J. Rieger, Stefanie Kreutmair, Weeda Mamozai, Wiebke Rösler, Philipp Hockl, Maiwand Ahmadsei, Sebastian M. Christ, Laura Motisi, Matthias Guckenberger, Dominik Schneidawind, Michael Mayinger

**Affiliations:** 1 Department for Medical Oncology and Hematology, University Hospital Zurich, Zurich, Switzerland; 2 Department for Medical Oncology and Hematology, University of Zurich, Zurich, Switzerland; 3 Department of Radiation Oncology, University Hospital Zurich, Zurich, Switzerland; 4 Institute of Experimental Immunology, University of Zurich, Zurich, Switzerland; 5 The LOOP Zurich, Zurich, Switzerland

**Keywords:** CAR T-cell therapy, Radiotherapy, Bridging therapy, Targeted therapy

## Abstract

**Background:**

Radiotherapy (RT) with immunochemotherapy (ICT) followed by CAR T-cell therapy may have synergistic effects due to cytoreduction and enhancing antigen spread, thereby inducing anti-cancer immune responses. The aim of this study was to analyze retrospective comparative data on the use of RT prior to anti-CD19 directed CAR T-cell therapy with a special focus on cytoreduction and RT related side effects.

**Methods:**

All patients aged ≥ 18 years with relapsed/ refractory Large B-Cell-lymphoma (r/r LBCL) treated with anti-CD19 CAR T-cell therapy in our institution from 05/ 2019–08/2023 were analyzed retrospectively, with the RT therapy group comprising all patients receiving RT with or without concomitant systemic therapy. The control (CO) group was manually matched on age, prior therapy lines and remission state at lymphodepletion. Post-RT tumor volumes (TV) were calculated for 6 out of 7 patients pre-CAR T and for 1 patient post-CAR T. Primary endpoints were reduction of TV and CAR T as well as RT related side effects. Secondary endpoints included overall survival (OS) and progression free survival (PFS).

**Results:**

8 patients receiving RT within 60 days prior to CAR T-cell infusion and 8 controls were included in the final analysis. 6 out of 8 patients received concomitant bridging therapy. RT alone or in combination with concomitant systemic therapy led to a significant reduction of TV (average reduction of 68%) within the radiated field from baseline to post RT (*p* = 0.028). The combination of RT and CAR T-cell therapy was not associated with an increased rate of CAR T related side effects or complications (cytokine release syndrome *p* = 0.6, immune effector cell-associated neurotoxicity *p* = 0.2, corticosteroid use *p* > 0.9, Tocilizumab use *p* > 0.9, transfer to intensive care unit *p* = 0.6). OS and PFS did not differ between the RT- and CO-group (OS *p* = 0.64, PFS *p* = 0.35).

**Conclusions:**

Our data indicate that RT is a feasible and effective way of cytoreduction before CAR T-cell therapy, also in combination with systemic chemotherapy.

**Clinical trial number:**

Not applicable.

**Supplementary Information:**

The online version contains supplementary material available at 10.1186/s13014-026-02822-z.

## Introduction

CAR T-cell therapy has revolutionized the treatment of relapsed/ refractory Large B-cell lymphoma (r/r LBCL) leading to sustained responses in previously refractory disease [1–4]. However, T-cell apheresis and CAR T-cell production consumes time and many patients require bridging therapy (BT, up to 60% in the Zurich CAR T-cohort) due to fast tumor progression and for symptom control [5, 6]. High tumor burden before CAR T-cell therapy is associated with an adverse outcome, reduction of tumor volume (TV) is therefore an imminent goal before lymphodepletion [7, 8]. In LBCL, BT to CAR T-cell therapy is most commonly performed through immunochemotherapy (ICT), including Polatuzumab-based regimen or radiotherapy (RT) [9]. In recent years, the relevance of RT in r/r LBCL with systemic disease diminished due to the increasing availability of potent systemic ICT. In the pre-CAR T-area, the use of RT in r/r LBCL indicated a potential benefit in specific patient populations [10–12]. RT induces cell death via a variety of mechanisms, including mitotic cell damage and interphase apoptosis within the tumor [13]. Furthermore, discussions are ongoing regarding a potential abscopal effect, which might lead to anti-tumor response outside of the RT-field through systemic immunomodulation [14]. Mechanisms regarding the potential effects of RT on immunomodulation were recently demonstrated in preclinical models, including pancreatic cancer and lymphoma mouse models, where it led to an enhanced antigen presentation and T cell-infiltration with improved CAR T-cell efficacy [15, 16]. This raises the question if RT together with ICT as a combined modality before CAR T-cell therapy may have beneficial synergistic effects, especially in high-risk patients with advanced stage disease and high TV.

The aim of this study was to retrospectively analyze the outcomes of RT prior to anti-CD19 directed CAR T-cell therapy with a special focus on cytoreduction and RT related side effects.

## Methods

### Data source and patient selection

We included all patients aged ≥ 18 years with r/r LBCL treated with anti-CD19 CAR T-cell therapy at the University Hospital Zurich from May 2019 to August 2023. The RT therapy group included all patients receiving external beam RT targeting r/r LBCL with or without concomitant systemic therapy. The control (CO)-group was manually matched to the RT group based on age, prior therapy lines and remission state at lymphodepletion. Original data were extracted from electronic medical records KISIM (CISTEC AG, Zurich, Switzerland) and ARIA (ARIA, Varian Medical Systems, Palo Alto, United States).

### Measurement of metabolic tumor volume based on FDG-PET/CT

Metabolic tumor volume (MTV) was quantified by importing FDG-PET/CT datasets into the ARIA oncology information system (Varian Medical Systems). Lesions were segmented using semi-automated, threshold-based tools within the clinical treatment planning environment, followed by manual refinement by experienced radiation oncologists, in line with the International Lymphoma Radiation Oncology Group (ILROG) guidelines [17].

### Endpoints

The primary endpoints were reduction of TV measured in cubic centimeters (CC), CAR T and RT related side effects as well as its complications. CAR T related side effects were documented and graded according to classification of Lee et al. [18]. RT related side effects were documented and graded using Common Terminology Criteria for Adverse Events (CTCAE) Version 5.0. Secondary endpoints were overall survival (OS) and progression free survival (PFS) after CAR T-cell infusion.

### Ethics approval

The investigation was approved by the Local Ethics Committee of the Medical Faculty of the University of Zurich, University Hospital Zurich (BASEC-Nr. 2023–02233). The project was conducted in accordance with the Declaration of Helsinki, and all patients provided general consent for scientific analysis. Patients refusing consent for scientific analysis were excluded.

### Statistical analyses

Comparisons between groups were analyzed using the Wilcoxon rank sum test (continuous data), or Fisher’s exact test (categorical data). OS and PFS were estimated using the Kaplan-Meier method, with the log-rank test being used to evaluate differences between groups. A p-value of *p* < 0.05 was considered statistically significant. Data were analyzed using R software version 4.3.3 (R Foundation for Statistical Computing, Vienna, Austria). Patients were excluded from the corresponding analysis in case of missing data.

## Results

### Patient characteristics and matching

**Fig. 1:**
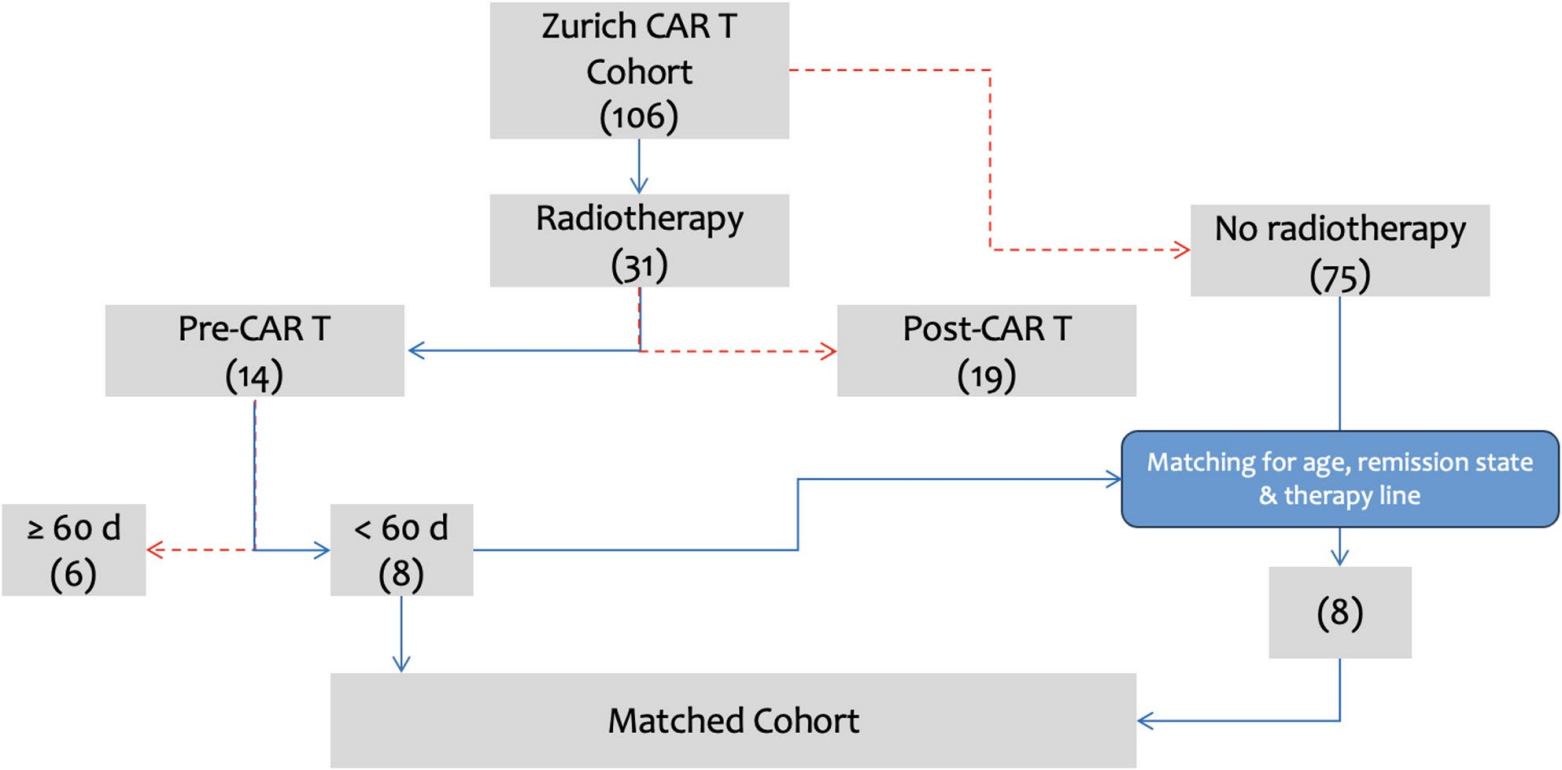
Patient selection and matching process

We screened 106 patients for our analysis, who received an anti-CD19 CAR T-product at our institution (Fig. 1). Of these 106 patients, 31 (31/106, 29%) received RT during their course of treatment and 14 (14/31, 45%) patients received RT before CAR T-cell therapy. A total of 8 (8/14, 57%) patients received RT within 60 days prior to CAR T-cell infusion and were included in the final analysis. In the next step, 8 patients out of the CO group were manually matched to the RT group, based on age, remission state at lymphodepletion and number of prior therapy lines.

The patient characteristics are shown in Table 1 and Supplementary. 38% (*n* = 3) of the included patients were female and 62% male (*n* = 5) in the RT group compared to an equal distribution (50% each) in the CO group (*p* > 0.9). The median age at retransfusion was 61 years in the RT group and 58 years in the CO group (*p* = 0.6). There were no statistically significant differences regarding the type of CAR T product, the number of prior treatment lines nor the remission state prior to CAR T-cell retransfusion (*p* > 0.9). The total TV tended to be higher in the RT group (624 cc vs. 236 cc), without reaching statistically significant difference (*p* = 0.22).

**Table 1:** Baseline characteristics

Characteristics	Groups		*P* – value
	Radiotherapy group *N* (%)	Control group *N* (%)	
**Sex**			> 0.9
Female	3 (38)	4 (50)	
Male	5 (62)	4 (50)	
**Age at Retransfusion**	61.3	58.2	0.6
**CAR T Product**			0.3
Lisocabtagene maraleucel	1 (12.5)	0 (0.0)	
Tisagene lecleucel	2 (25.0)	5 (62.5)	
Axicabtagene ciloleucel	5 (62.5)	3 (37.5)	
**Number of Prior Treatment Lines**			> 0.9
1	0 (0.0)	0 (0.0)	
2	2 (25.0)	2 (25.0)	
3	3 (37.5)	3 (37.5)	
4	3 (37.5)	3 (37.5)	
**Remission prior to CAR T-cell therapy**			> 0.9
Partial Remission	1 (12.5)	1 (12.5)	
Progressive Disease	7 (87.5)	7 (87.5)	
**Total tumor volume (mean in cc)**			0.22
	624 cc	236 cc	

### Radiotherapy application and efficacy

An interdisciplinary tumor board decided on treatment with RT. All RT patients underwent CT simulation. The treated target included a clinical target volume (CTV) defined based on lesion expansion in PET/CT and/or MR imaging respectively and expanded geometrically by 3 to 10 mm to form the planning target volume (PTV). Patients were treated daily as shown in Table 2. All patients were treated in VMAT technique. Post-RT TV was estimated pre-CAR T except for patient #4, in which case no post-RT pre-CAR T staging was available. Post-RT TV for patient #3 was not available for analysis due to rapid progress and death. Prior to RT, 5 of 8 patients were symptomatic. All symptoms improved after RT with 3 patients reporting a complete response of symptoms 2 weeks after RT. The mean time interval between the last radiotherapy application and the subsequent PET/CT scan was 9 days.

**Table 2:** RT treatment characteristics

ID	Dose	Location	Days from RT to CAR T infusion	Volume RT (cc)	Volume not treated (cc)	Concomitant Therapy	Treated bulk symptomatic
1	10 × 3 Gy	Intracranial bulk	28	43	0	Ibrutinib (before/ after RT)	-
2	7 × 4 Gy	Left jugular foramen	16	2.4	2243.5	Blinatumomab (before RT), Intrathecal chemotherapy (after RT)	Dysphagia, increased salivation
3	1 × 8 Gy	Cervical right	4	609.3	607.6	None	Dyspnea
4	5 × 4 Gy	Left supraclavicular/Mediastinum	12	557	23	Polatuzumab, Rituximab, Bendamustine (before RT)	-
5	10 × 3 Gy	Mediastinum	36	131.6	0	Pembrolizumab (before RT)	Pain
6	5 × 4 Gy	Left hip	28	303	36	None	Pain
7	1 × 8 Gy + 5 × 2 Gy; 5 × 4 Gy	Right pelvis Right thigh	33	262.1	3.3	Rituximab, Ifosfamide, Cyclo-phosphamide, Etoposide (before RT)	Pain
8	15 × 2 Gy	Mediastinum	50	173.4	0	Venetoclax (before RT), Pembrolizumab (after RT)	-

**Fig. 2:**
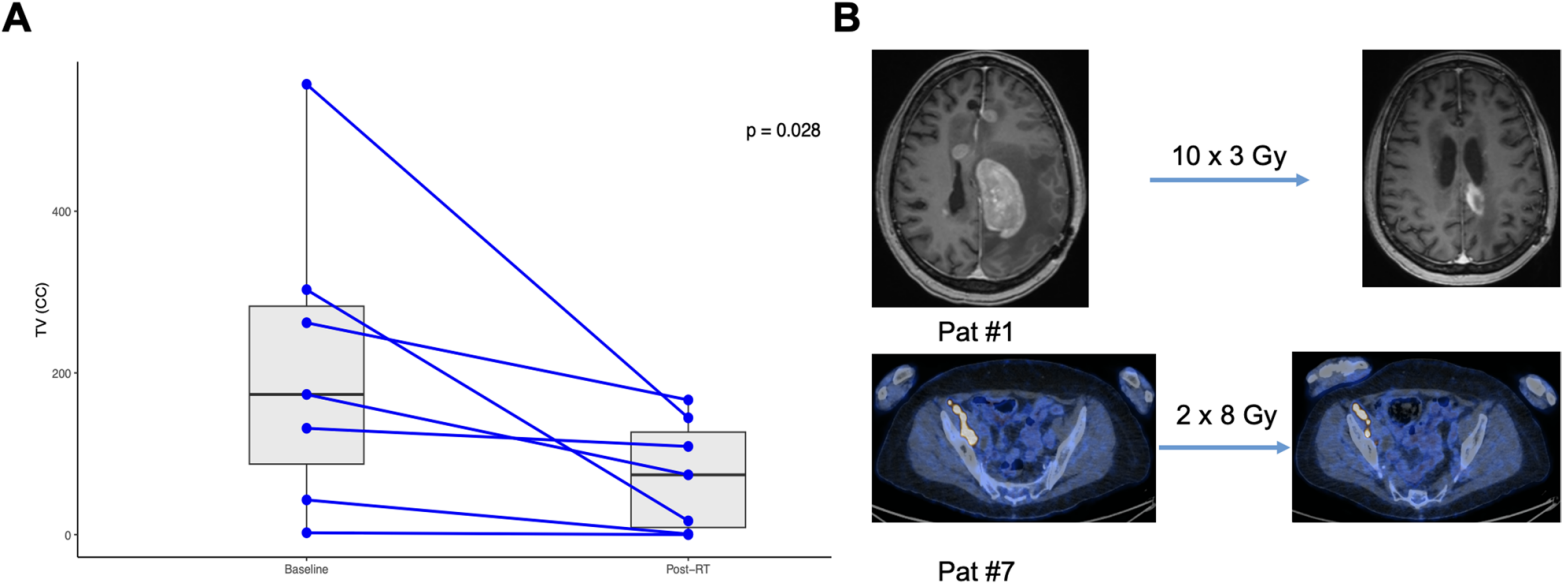
**A**) In-field cytoreduction after the application of RT. **B**) Example of the tumor volume reduction in two selected patients

The use of RT alone or in combination with concomitant systemic therapy led to a significant reduction (average reduction of 68%) in tumor volume in the RT group within the irradiated field from baseline to post-RT (Fig. 2; *p* = 0.028). The effect remained statistically significant even after exclusion of patient #4 due to missing TV estimation pre-CAR T (*p* = 0.04).

### Radiotherapy and CAR T related side effects

**Fig. 3:**
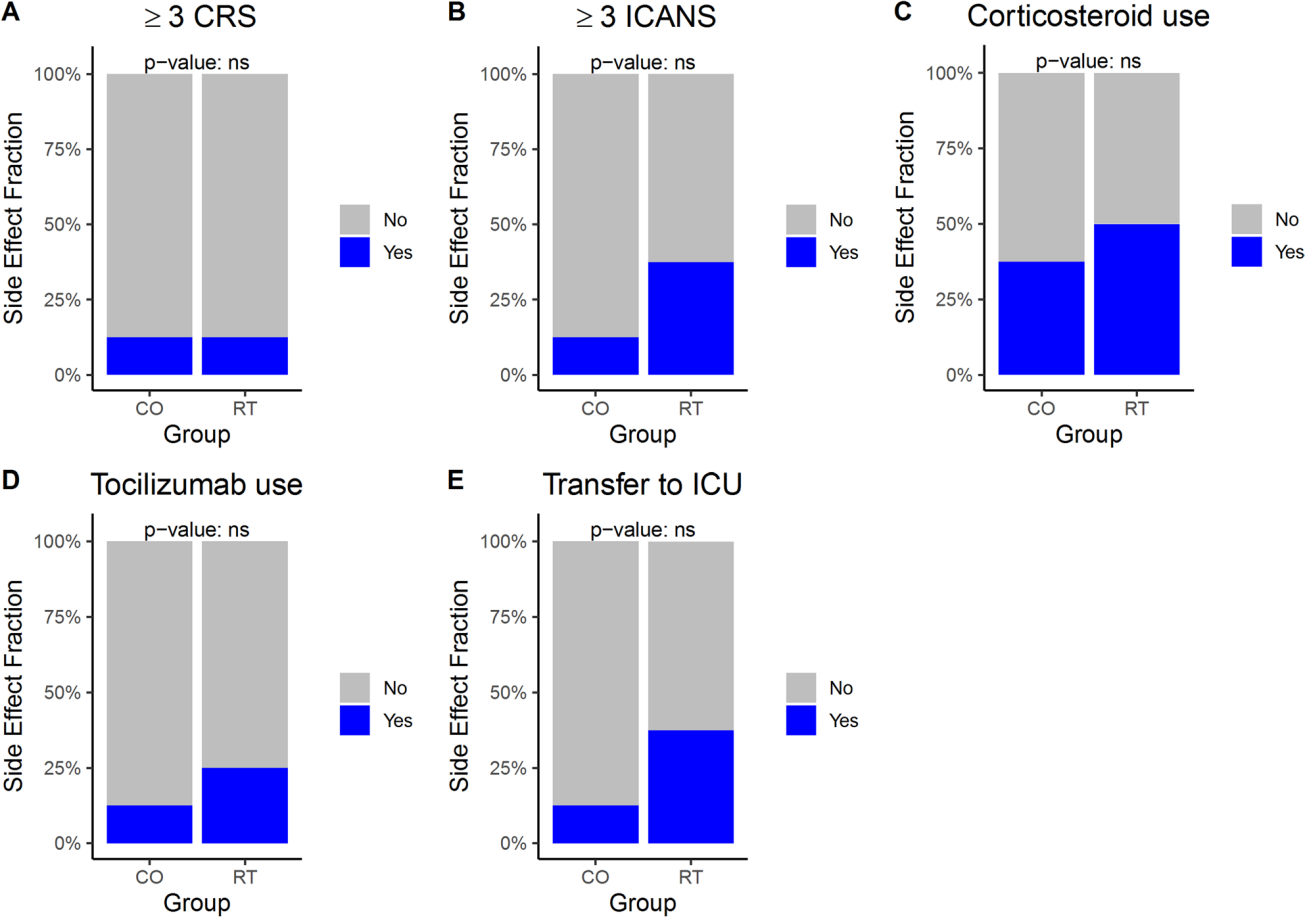
CAR T related side effects and therapeutical consequences for the corresponding event. Abbr.: Cytokine-release syndrome (CRS), immune effector cell-associated neurotoxicity (ICANS), intensive care unit (ICU)

The combination of RT and CAR T-cell therapy did not lead to an increased rate of CAR T related side effects compared to the CO group (CRS *p* = 0.6, ICANS *p* = 0.2, corticosteroid use *p* > 0.9, Tocilizumab use *p* > 0.9, transfer to intensive care unit (ICU) *p* = 0.6; Fig. 3). The rate of severe CRS or ICANS, respectively the use of corticosteroids or Tocilizumab did not significantly differ between both groups.

**Fig. 4:**
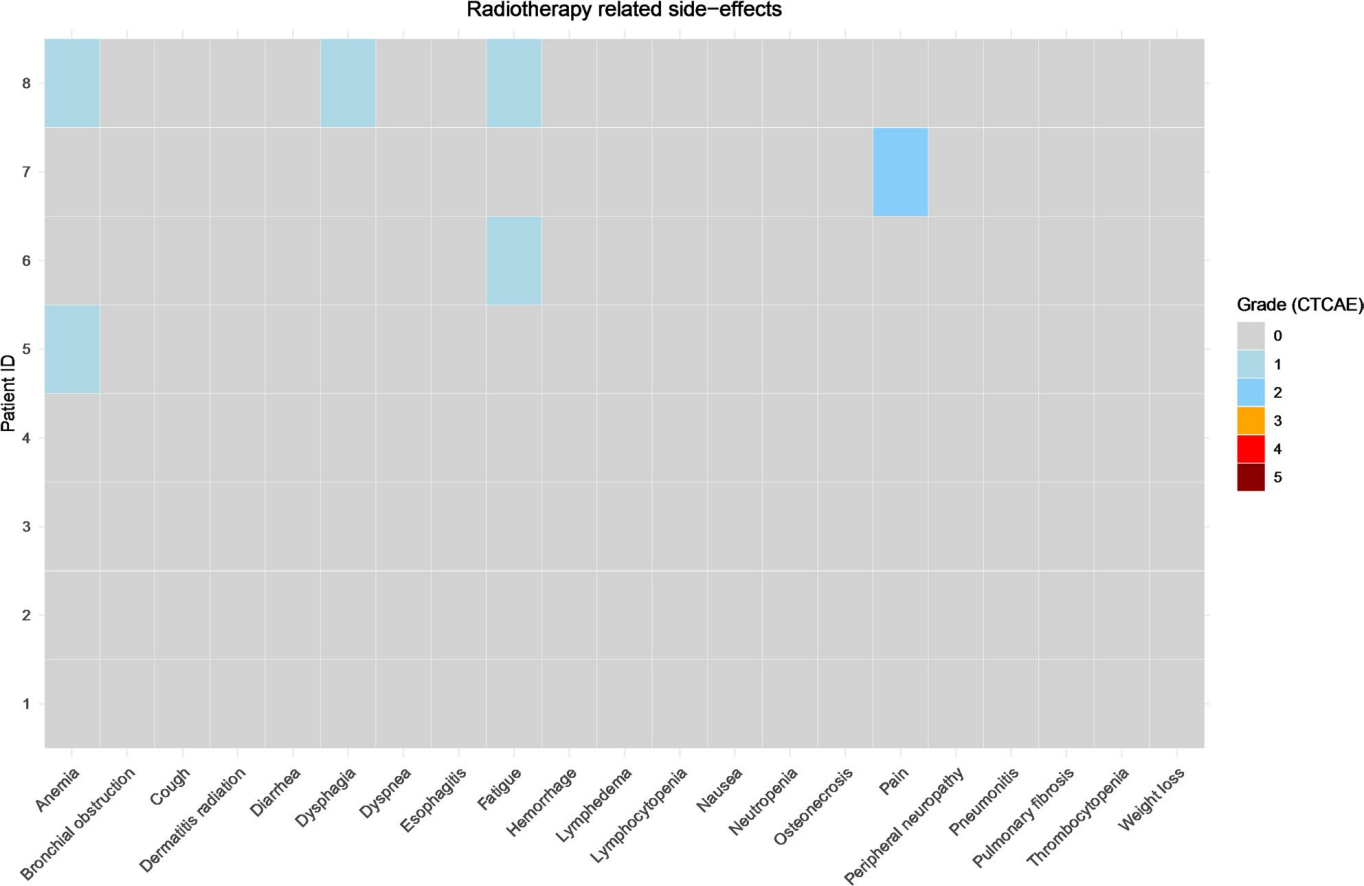
RT related side effects according to CTCAE 4.0 grading

In the next step, we controlled for RT related side-effects in the RT group. We detected only a low number of clearly RT related events. Most patients (6/8) received a concomitant chemotherapy (Table 2). Most common side effects were fatigue (*n* = 2) and anemia (*n* = 2) followed by pain within the radiation field (*n* = 1) and dysphagia after RT (*n* = 1). No patient experienced a CTCAE ≥ 3 event (Fig. 4).

### Overall survival and progression free survival

OS and PFS over time did not demonstrate any statistically significant differences between the RT- and CO-group (Log Rank OS *p* = 0.64, Log Rank PFS *p* = 0.35). OS 24 months after CAR T-cell retransfusion was 45% (95% confidence interval; 20% – 100%) in the RT group vs. 63% (95% confidence interval; 37% – 100%) in the CO-group (*p* = 0.5). Median follow-up was 556 days in the RT-group, while it was not reached in the CO-group. PFS at 24 months after the application of the CAR T product was 38% in the RT group (95% confidence interval; 15% – 92%) compared to 50% in the CO-group (95% confidence interval; 25% – 100%, *p* = 0.6). Median time of follow up was 611 days in the CO-group and 881days in the RT-group.

## Discussion

In this retrospective single center comparative analysis, 8 patients received RT within 60 days prior to CAR T-cell infusion and 8 controls were included in the final analysis. RT led to a significant reduction of TV within the irradiated field from baseline to post RT with an average decrease of 68%. Six out of eight patients received concomitant systemic therapy. The combination of RT and CAR T-cell therapy was not associated with an increased rate of CAR T related side effects in our cohort. OS and PFS did not differ between the RT and CO group. The significantly reduced TV in the setting of CAR T-cell therapy provides additional insight, as the exact extent of cytoreduction by RT is not routinely assessed in this setting. Furthermore, RT with or without systemic chemotherapy resulted in a tolerable side effect profile showing no increase in CAR T-cell specific adverse events compared to the CO group. The use of RT in the setting of CAR T-cell therapy has initially been regarded with some reservations due to potential concerns of delaying CAR T-cell therapy or augmenting therapy related side effects. Only 7% (8/106) of the patients at our institution received RT within 60 days prior to CAR T-cell therapy, likely reflecting the above-mentioned concerns, especially when combined with additional systemic chemotherapy. However, our data together with recent publications did not confirm this concern [9, 19–21]. RT bridging (in combination with systemic therapy) prior to CAR T-cell therapy has been reported in both prospective and retrospective analyses and appears to constitute a safe and feasible treatment option, providing acceptable toxicity profiles while resulting in cytoreduction and improved PFS (Table 3).

**Table 3:** Selected investigations reporting on RT bridging prior to CAR T-cell therapy

Trial	Year	Study type	Number of RT BT patients	Histology	Main RT related observation
Ababneh et al. [22]	2025	Prospective	10	DLBCL, TFL, HGBCL	Safety and feasibility of weekly adaptive BRT
Yegya-Raman et al. [23]	2025	Retrospective	172	LBCL, MCL, BL	Acceptable toxicity profile with favorable clinical outcomes when compared to historical controls
Cederquist et al. [24]	2024	Retrospective	12	CNS B-cell lymphoma	CNS RT achieved 74% mean reduction in lesion size 12 days prior to CAR T-cell infusion
Roddie et al. [9]	2023	Retrospective	62	B-cell lymphoma	No increased side effects for patients treated with RT. BRT response highest in patients treated with RT (73% vs. 40% in CT/CMT groups)
Fan et al. [20]	2022	Retrospective	20	DLBCL	RT in combination with CAR T-cells results in no high-grade toxicities
Hubbeling et al. [21]	2022	Retrospective	40	DLBCL, MCL, BL	BRT produced cytoreductions in diameter, SUV, MTV, and LDH
Pinnix et al. [25]	2020	Retrospective	11	B-cell lymphoma	Improved PFS for RT-bridged compared with ST-bridged patients

*Abbr.*: DLBCL = diffuse Large B-Cell Lymphoma; TFL = Transformed Follicular Lymphoma; HGBCL = high-grade B-Cell Lymphoma; LBCL = Large B-cell Lymphoma, MCL = Mantle Cell Lymphoma, BL = Burkitt Lymphoma, BRT = bridging radiotherapy; SUV = standardized uptake value; MTV = metabolic tumor volume; LDH = lactate dehydrogenase

Our study has several limitations. First, it is a retrospective single center analysis with a limited number of patients, making it susceptible to potential bias and limiting its generalizability. Secondly, most patients in the RT group received a concomitant systemic chemotherapy, complicating the assignment of side-effects or therapy success. Lastly, although we performed a matched analysis, the patients in the CO group received different CAR T products and the CO group tended to have a lower total TV before CAR T-cell therapy than the RT group. Therefore, our analysis might underestimate the effect of the cytoreduction in CAR T-cell regarding OS and PFS in our patients.

In summary, our data suggest that RT is a feasible and potentially effective approach for cytoreduction prior to CAR T-cell therapy, including when used in combination with concomitant systemic therapy.

## Supplementary Information

Below is the link to the electronic supplementary material.

Supplementary Material 1

## Data Availability

Data can be made available upon reasonable request to the author and as far as possible regarding the ethical approval.
