# Supplementary material for: Bridging radiotherapy before anti-CD19 CAR T-cell therapy for Large B-cell lymphoma – results from a single-center study
Source: Radiat Oncol. 2026 Mar 31;21:69. doi: 10.1186/s13014-026-02822-z (PMC13154906; doi:10.1186/s13014-026-02822-z)
Supplement: Supplementary file 1 — Supplementary Material 1 [file 13014_2026_2822_MOESM1_ESM.docx]

S1: PET/CT findings description

| ID | Last PET-CT before CAR T | PET-CT details |
| --- | --- | --- |
| RT1 | LS NE (PD) | Intracranial manifestation (no peripheral manifestation) |
| RT2 | LS5 (PD) | Nodal lesions infradiaphragmal, extranodal lesion |
| RT3 | LS5 (PD) | Nodal lesions supra- and infradiaphragmal, extranodal lesions |
| RT4 | LS5 (PD) | Nodal lesions supra- and infradiaphragmal, extranodal lesion |
| RT5 | LS5 (PD) | Nodal lesions supradiaphragmal |
| RT6 | LS5 (PD) | Nodal lesions supra- and infradiaphragmal, extranodal lesions |
| RT7 | LS5 (PD) | Nodal lesions supra- and infradiaphragmal |
| RT8 | LS4 (PR) | Nodal lesions supradiaphragmal |
| CO1 | LS NE (PR) | Extranodal lesion |
| CO2 | LS5 (PD) | Nodal lesions supra- and infradiaphragmal, extranodal lesion |
| CO3 | LS5 (PD) | Nodal lesions infradiaphragmal |
| CO4 | LS5 (PD) | Nodal lesions infradiaphragmal, extranodal lesion |
| CO5 | LS5 (PD) | Nodal lesions supradiaphragmal, extranodal lesions |
| CO6 | LS5 (PD) | Nodal lesions supra- and infradiaphragmal |
| CO7 | LS5 (PD) | Nodal lesion (bulk) infradiaphragmal, extranodal lesions |
| CO8 | LS 5 (PD) | Nodal lesions infradiaphragmal, extranodal lesions |

S2: Control group treatment details

| ID | Days from PET-CT to CAR T-cell retransfusion | Systemic therapy in the pre-CAR T-cell setting | Therapy between PET-CT and CAR T-cell retransfusion |
| --- | --- | --- | --- |
| CO1 | 13 | Polatuzumab, Bendamustin, Rituximab  (2 cycles) | Yes  (1 cycle) |
| CO2 | 18 | Polatuzumab, Bendamustin, Rituximab  (2 cycles) | No |
| CO3 | 4 | Rituximab, Ifosfamide, Carboplatin, Etoposide  (3 cycles) | No |
| CO4 | 13 | Polatuzumab, Bendamustin, Rituximab  (2 cycles) | No |
| CO5 | 20 | Rituximab, Gemcitabine  (1 cycle)  Methotrexate  (1 cycle) | No |
| CO6 | 10 | Polatuzumab, Rituximab (1 cycle) | No |
| CO7 | 8 | Gemcitabine, Oxaliplatin  (1 cycle) | No |
| CO8 | 8 | Carmustine, Etoposide, Cytarabine, Melphalan | No |

S3: Specific treatment details of selected patients in the RT group

| ID | Status | Prior therapy lines | Additional information |
| --- | --- | --- | --- |
| RT3 | RT without concomitant therapy | 1L: R-CHOP  2L: R-ICE  Bridging: R-ICE, R-DHAOx | Refractory to CAR T-cell therapy; died of progressive disease after retransfusion |
| RT4 | Progression Free | 1L: R-CHOP  Salvage: R-DHAP  Bridging: R-Pola-BR | Progression free after CAR T-cell therapy |
| RT5 | Progression Free | 1L: R-DA-EPOCH  Salvage: R-DHAOx  Bridging: Pembrolizumab | Progression free after CAR T-cell therapy |
| RT6 | RT without concomitant therapy/  Progression Free | 1L: R  2L: R-CVP | Progression free after CAR T-cell therapy |
